# Internal validation strategy for high dimensional prognosis model: A simulation study and application to transcriptomic in head and neck tumors

**DOI:** 10.1016/j.csbj.2025.08.035

**Published:** 2025-09-03

**Authors:** Antoine Dubray-Vautrin, Victor Gravrand, Grégoire Marret, Constance Lamy, Jerzy Klijanienko, Sophie Vacher, Ladidi Ahmanache, Maud Kamal, Olivier Choussy, Nicolas Servant, Célia Dupain, Christophe Le Tourneau, Jimmy Mullaert

**Affiliations:** a Institut Curie, INSERM, Saint Cloud U1331, France; b Department of surgical oncology, Institut Curie, Paris, France; c Genetics Department, Institut Curie, Paris, France; d Department of Drug Development and innovation (D3i), Paris, France; e Department of pathology, Institut Curie, Paris, France; f Université Paris-Saclay, UVSQ, Versailles 78000, France

**Keywords:** Transcriptomic, Prognosis, Simulation, Head and neck, Penalized regression, Validation

## Abstract

**Background:**

Predictive models using high-dimensional data, such as genomics and transcriptomics, are increasingly used in oncology for time-to-event endpoints. Internal validation of these models is crucial to mitigate optimism bias prior to external validation. Common strategies include train-test, bootstrap, and (nested) cross-validation. However, no benchmark exists for these methods in high-dimensional settings. We aimed to compare these strategies and provide recommendations in the field of transcriptomic analysis.

**Method:**

A simulation study was conducted using data from the SCANDARE head and neck cohort (NCT 03017573) including n = 76 patients. Simulated datasets included clinical variables (age, sex, HPV status, TNM staging), transcriptomic data (15,000 transcripts), and disease-free survival, with a realistic cumulative baseline hazard. Sample sizes of 50, 75, 100, 500, and 1000 were simulated, with 100 replicates each. Cox penalized regression was performed for model selection, followed by train-test 70 % training), bootstrap (100 iterations), 5-fold cross-validation, and nested cross-validation (5 ×5) to assess discriminative (time-dependent AUC and C-Index) and calibration (3-year integrated Brier Score) performance.

**Results:**

Train-test validation showed unstable performance. Conventional bootstrap was over-optimistic, while the 0.632 + bootstrap was overly pessimistic, particularly with small samples (n = 50 to n = 100). The k-fold cross-validation and nested cross-validation improved performance with larger sample sizes, with k-fold cross-validation demonstrating greater stability. Nested cross-validation showed performance fluctuations depending on the regularization method for model development.

**Conclusion:**

The K-fold cross-validation and nested cross-validation are recommended for internal validation of Cox penalized models in high-dimensional time-to-event settings. These methods offer greater stability and reliability compared to train-test or bootstrap approaches, particularly when sample sizes are sufficient.

## Graphical Abstract

**Figure:**
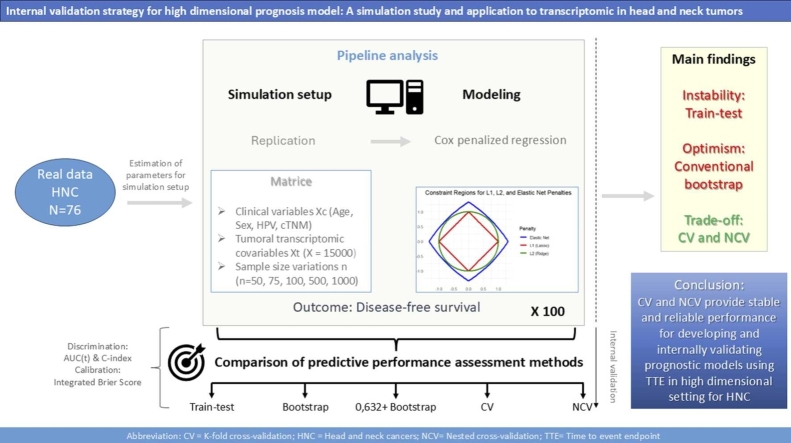

## 1 Introduction

Multidimensional research has been rapidly evolving since the advent of genomic and transcriptomic sequencing at the end of the 2000s [1]. From the emergence of next generation sequencing (NGS) and development of targeted molecular therapies, therapeutic decision-making processes for many cancer types have become increasingly complex due to the inclusion of molecular biomarkers in prognostication [2]. Predictive models are important tools to provide estimates of patient outcomes [3], but developing such models is particularly challenging when dealing with high-dimensional data such as genomics, epigenomics, transcriptomics, proteomics, metabolomics because the large number of variables relative to the number of samples can lead to overfitting, increased noise, and instability in the results. This complexity often reduces model generalizability and makes it difficult to identify truly informative features, thereby complicating model development and interpretation.

Model development aims to detect the relevant features and discard irrelevant ones. Selecting prognostic variables in high-dimensional data is challenging due to correlations between covariates, limited statistical power, and increased risk of type I errors in variable selection with prognosis outcome when using multi-testing approaches. Several methods could be used for selection, such as Cox penalized regression or machine learning approaches [4] with time-to-event endpoints. Penalized Cox regression models, such as LASSO (Least Absolute Shrinkage and Selection Operator) and elastic net, exhibit good sparsity and interpretability [5] for time-to event outcomes. Penalized regression is performed to estimate the prognostic importance of biological factors and to assess the correlation between the variables integrating all available data to compute more comprehensive models.

The validation of a statistical model aims to assess its performances without bias [6]. Internal validation procedures evaluate the model using the same dataset used for its development, which helps estimate how well the model fits the training data. In contrast, external validation involves testing the model on an independent dataset to assess its generalizability to new, unseen data. Although external validation is crucial for confirming the robustness and applicability of prognostic models [7], the assessment of the model performance particularly in high dimensional setting using time-to event endpoints is notably affected by optimism and instability because of right censoring [8]. Commonly used approaches for internal validation include train-test, bootstrap, cross-validation and nested cross-validation. Train-test validation of clinical prediction models which divides data into training and testing sets, may reduce predictive accuracy and the precision of validation in prognosis studies [9]. Bootstrap validation methods are also performed to validate penalized models but carry a risk of overestimation depending on the bootstrap estimator; correction methods have been described for linear and binary models to limit optimism bias [10], [11]. The 0.632 + bootstrap estimator, which combines the resampling error and bootstrap error with a weighting scheme to reduce bias, has been shown to produce less optimism bias, particularly in non-regularized models using time-to-event (TTE) endpoints, where outcomes are measured as the time until an event such as death or relapse occurs [12]. K-fold cross-validation seems to provide a good balance between bias and stability in high dimensional setting [13], [14] but it has been poorly evaluated using time-to-event endpoints. Nested-cross validation is also performed in small sample datasets, optimizing hyperparameters with good accuracy; it aims to combine both selection and validation of the best model [15], [16] within the same procedure but has been under-evaluated using time-to-event endpoints in high-dimensional settings. To date, no studies have directly compared the performance of internal validation methods in omics approaches, particularly those involving the integration of transcriptomic data using time to-event endpoint.

The aim of the study is therefore to compare the performance of train-test, bootstrap, cross validation and nested cross validation with transcriptomic data using time-to event endpoints in a head and neck carcinomas simulation study.

## 2 Methods

### 2.1 Simulation study and implementation

**Fig. 1:**
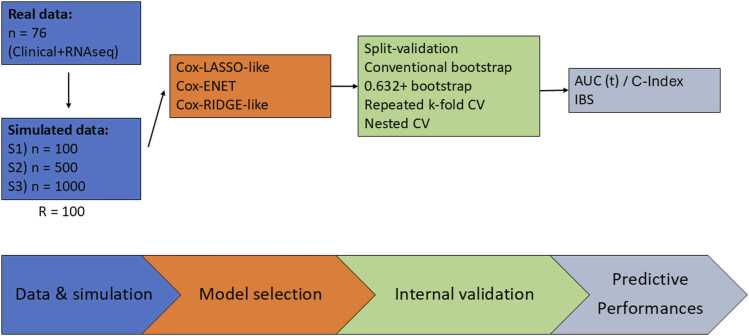
Flow chart of statistical process of the simulation.

Datasets of different sample size (50, 75, 100, 500, and 1000), inspired by head and neck oncology, were generated, with r = 100 replicates per scenario. An independent dataset of n = 1000 samples was simulated with the same approach as the replicates. All simulation and analysis were performed with R software (version 4.4.0). The entire process of the study is summarized in Fig. 1.

### 2.2 Data generating mechanism

#### 2.2.1 Data

The original data from the SCANDARE study (NCT03017573) were collected prospectively from 2017 to 2020 as part of a biobanking initiative in cancer patients. The primary objective was to better understand the interplay between molecular alterations within the tumor, the tumor microenvironment, and the immune response.

SCANDARE is a multicenter, prospective biobanking study collecting tumor samples (with or without nodal tissue), plasma, and blood at various time points. The study included patients with several cancer types: ovarian cancer, triple-negative breast cancer, head and neck cancer, advanced-stage treatment-naïve cervical cancer, sarcomas (including breast angiosarcoma and uterine sarcoma). This study specifically focused on the head and neck cancer population.

Participants met the following criteria: newly diagnosed and treatment-naïve patients eligible for surgical intervention, age ≥ 18 years, both male and female, and signed informed consent.

A multi-platform approach was applied to tumor and blood samples, including: pathological evaluation, genomic profiling, transcriptomic analysis, DNA methylation assessment. These platforms enabled a comprehensive characterization of tumor biology and host immune features in the selected population.

#### 2.2.2 Clinical data simulation

We simulated 4 independent covariables: *age*, *sex*, *HPV status* and *TNM staging*. Age was sampled using a normal distribution with mean of 65 years and a standard deviation of 10 years. We simulated *Sex* and *HPV status* using a Bernoulli distribution p = 0.3 for both. We sampled the *TNM staging* (I to IV) with probabilities of 0.22, 0.13, 0.25 and 0.40, based on SCANDARE head and neck cohort (NCT03017573).

#### 2.2.3 Transcriptomic data simulation

We independently simulated log-transformed count data for 15000 transcripts bases on SCANDARE head and neck distribution and dispersion parameters independently. We applied a four-step simulation: (i) the simulation of the mean expression µ_1_ of 15 000 transcripts using a normal distribution with µ_0_ = 2.3 and σ_0_ = 1.8, (ii) a dispersion parameter τ_1_ following a lognormal distribution with µ_τ_ = 2.8 and log(σ_τ_) = 0.4. (iii) the simulation of value of each sample (n) of the datasets using a skewed normal distribution with mean µ_2_ = exp(µ_1_), standard deviation σ_2_ = (exp(µ_1_)/1.3) x τ_1_ and skewness ϒ = 2; (iv) we replicated this simulation 100 times for each sample size n = 50, 75, 100, 500 and 1000. We represented the logarithm of the variance according to the logarithm of the mean of the count in TPM (Transcript Per Million) in supplementary data (eFigure 1) for simulated data and original data from SCANDARE.

#### 2.2.4 Time-to-event simulation

We estimated the coefficients used for clinical covariables (eTable 1) based on the SCANDARE head and neck cancer cohort with a non-penalized multivariable Cox regression. The coefficients β_Age_, β_Sex_, β_TNM_, β_HPV_ were respectively, 0.02, −0.8, 0.3 and −0.5. For TNM, a linear relation with the log hazard was assumed and only one coefficient was needed. For each dataset, we assumed that 200 out of a total of 15 000 transcripts are associated with the risk of cancer recurrence through a Cox regression model with 100 coefficients generated from an uniform U(-0.1,-0.01) distribution and 100 coefficients generated from an uniform U(0.01,0.1) distribution randomly affected to the logarithm of the count. The coefficients for the transcriptomics covariates were applied on a logarithmic continuous scale.

We generated individual event times [17] according to the survival model using the estimated cumulated baseline hazard *H*_0_(*t*) of the SCANDARE head and neck cohort and the coefficients *β*xi for time-to event distribution simulation with the inverted method:

$$\mathrm{Ti}=H_{0}^{-1}\left[-\log\left(U\right)\times \exp\left(-\beta x_{i}\right)\right]$$

Where *Xi*= (*Xi*1,…,*Xip*) *T* the vector of covariates for patient i and *β*= (*β*1,…,*βp*) the vector of associated coefficients.

The data generating mechanism was mechanism was consistent across all 100 replicates for each scenario (n = 50, 75, 100, 500 and 1000) as well as for the independent dataset (n = 1000).

### 2.3 Outcome

In this study, we selected disease-free survival (DFS) as the outcome of interest for our statistical analysis. DFS was defined as the time from the start of treatment until death from any cause or local, regional, or metastatic recurrence, which was coded as event 1 when it occurred. Time horizons of 3 and 5 years were fixed for the dynamic simulation.

### 2.4 Model development

The model development had to measure the individual probability of recurrence or death assed by the DFS. For each datasets, we fitted a Cox model with elastic net penalty with a parameter α equal to 0.05 (ridge-like penalty), 0.5 (elastic net) and 0.95 (lasso-like penalty). The penalty λ was set using ten-fold cross-validations using cv.glmnet function of glmnet r package and we chose the $\lambda_{\left(1\cdot \mathrm{SE}\right)}$ to select best model in entire dataset using all visits before internal validation testing [10].

### 2.5 Internal validation method

The train-test validation was implemented by randomly dividing the original dataset into two subsamples: a training set (used for model derivation) and a testing set (used for model validation). The individual predicted survival probabilities were estimated on the testing set. We also employed the conventional bootstrap procedure estimator [3], [9], using a fixed number of iterations. In this approach, the training set is the bootstrap sample, and the testing set is the original dataset for each iteration, with performance metrics evaluated on the testing set. Additionally, we implemented the 0.632 + bootstrap estimator described by Efron and Tibshirani [18].

For the k-fold cross-validation, as commonly performed in the literature [14], the dataset was divided into K equal-sized subsamples. K−1 subsamples were used as the training set, and the remaining subsample served as the testing set for assessing performance metrics. This process was repeated for each of the K subsamples. Lastly, we employed nested cross-validation as described in the literature [19], [20]. This method involves tuning the parameter $\lambda_{\left(1\cdot \mathrm{SE}\right)}$ within inner loops to optimize the model, followed by applying the optimized model on the outer testing set to evaluate predictive performance. This procedure was repeated multiple times across the K outer folds.

For all methods, the optimal $\lambda_{\left(1\cdot \mathrm{SE}\right)}$ was determined using ten-fold cross-validation on the training set with the ‘glmnet’ function prior to performing the validation procedure. The specific parameters used for each method of internal validation were:

i. Train-test partition: The dataset was randomly split into training (70 %) and testing (30 %) sets. Individual predictions were calculated on the testing set.
ii. Conventional Bootstrap: After determining $\lambda_{\left(1\cdot \mathrm{SE}\right)}$ 100 bootstrap iterations were performed. Predictions were generated using the conventional bootstrap method [3], [12], [21] with timeROC and pec R packages.
iii. 0.632 + Bootstrap: After determining $\lambda_{\left(1\cdot \mathrm{SE}\right)}$, 100 bootstrap iterations were performed. Predictions were obtained using the 0.632 + bootstrap estimator, and performance was evaluated with timeROC and pec R packages.
iv. K-fold-cross validation: We performed K-fold cross-validation with $\lambda_{\left(1\cdot \mathrm{SE}\right)}$ optimization. We fixed a K= 5 and 10 repetitions and obtained the prediction using the test samples of each fold and repetitions.
v. Nested cross-validation: We performed 5-repeated nested cross-validation with K= 5 outer loop segmentation and a n = 5 inner loop segments. About the outer Loop, the dataset was divided into K=outer folds. In each iteration of the outer loop, one fold was set aside as the testing set, while the remaining K−1 folds served as the training set. About the inner loop, within the training set of the outer loop, the inner loop was performed with *J*= 5 folds. These inner folds were used to tune the hyperparameter $\lambda_{\left(1\cdot \mathrm{SE}\right)}$ using cross-validation. Specifically, in each iteration of the inner loop, J−1 folds were used to train the model, and the remaining fold was used to validate and evaluate the performance for hyperparameter tuning. The "best model" refers to the model trained on the entire training set of the outer loop using the optimal $\lambda_{\left(1\cdot \mathrm{SE}\right)}$ determined from the inner loop. This best model was then applied to the testing set of the outer loop to compute individual predictions and evaluate the performance metrics.

An independent validation of the best models after internal validation process was performed using the independent independent simulated data (n = 1000) not used in the internal validation process to assess the predictive performance.

### 2.6 Estimands and performance metrics

We assessed the predictive performances of the estimated models with the time dependent area under the curve (AUC) and the C-index as measures of model discrimination. We considered for each dynamic analysis of AUC, a cumulative/dynamic method for calculation of AUC-time dependent [22], [23], [24]. The AUC ranges from 0.5 (uninformative model) to 1 (perfect discrimination). The time-dependent cumulative/dynamic AUC between time 0 and a given horizon time is obtained from the timeROC R package, as the mean of the 100 replicates for each scenario. Horizon times ranged from 1 to 5 year with 0.1 year increment and a standard deviation estimate across all the replicates at 3 year time. The C-index and corresponding standard deviation were estimated using the R package survcomp. To compare the variance of the 3-year AUC across replicates between the internal validation strategies, we conducted a homogeneity of variance test using Bartlett’s test with the null hypothesis of equal variances. If the p-value was less than 0.05, adjusted pairwise comparisons using Fisher’s test were conducted. Pairwise equality of variance indicates comparable stability between two methods according to the model development approach and sample size.

We also evaluated calibration performances using integrated Brier Score (IBS) estimated with inverse probability of censoring weights (IPCW)[25], [26] calculating the individual survival probabilities using pec package from time 0–3 years.

#### 2.6.1 Measure of bias

We obtained the oracle AUC after calculating linear predictors using the coefficients generated for data simulation with an independent simulated dataset. We developed the naïve 3 years-IBS using the same linear predictors as the oracle AUC calculation. The oracle performances represent the best possible model outcomes, obtained by using all true coefficients from the simulation process. This oracle model serves as a benchmark that should not be surpassed. We used an independent validation approach with the independent simulated dataset not used for the internal validation strategies measuring the AUC(t) and the 3 year-IBS with the hyperparameter of the models priorly fixed before internal validation (α and $\lambda_{\left(1\cdot \mathrm{SE}\right)}$) and into the nested-cross validation approach.

We obtained the measure of optimism bias by calculating the difference between the oracle AUC model and the mean of AUC obtained by each strategy of internal validation: train-test validation, conventional bootstrap, 0.632 + bootstrap, k-fold cross-validation, and nested cross-validation.

#### 2.6.2 Application

We assessed the internal validation methods, train-test validation, conventional bootstrap method repeated-K-fold cross validation and nested cross-validation on SCANDARE clinical and transcriptomic original and real dataset with n = 76 samples. We developed Lasso-like regularization with α= 0.95 using $\lambda_{\left(1\cdot \mathrm{SE}\right)}$ optimization on the entire dataset incorporating clinical covariables (Ag, Sex, HPV status, TNM staging) and 14924 transcriptomics variables using logarithmic transformation of the TPM normalization. We measured both AUC(t) and the 3-year IBS to evaluate performance of all method of internal validation.

## 3 Results

In this benchmark study we presented the performances of internal validation strategy by model development regularization according to discriminative performance and calibration performance. We reported on eTable 1 the non-convergence rate after model selection by regularization of penalized regression.

### 3.1 Time-dependent discriminative performance

We represented 3-year AUC on Table 1. The mean of 3-year AUC of independent validation according to sample size were: 0.50 [0.48–0.53] (n = 100), 0.55 [0.50–0.58] (n = 500) and 0.60 [0.57–0.63] (n = 1000). The oracle 3 year-AUC using the parameters of simulation was 0.82.

**Fig. 2:**
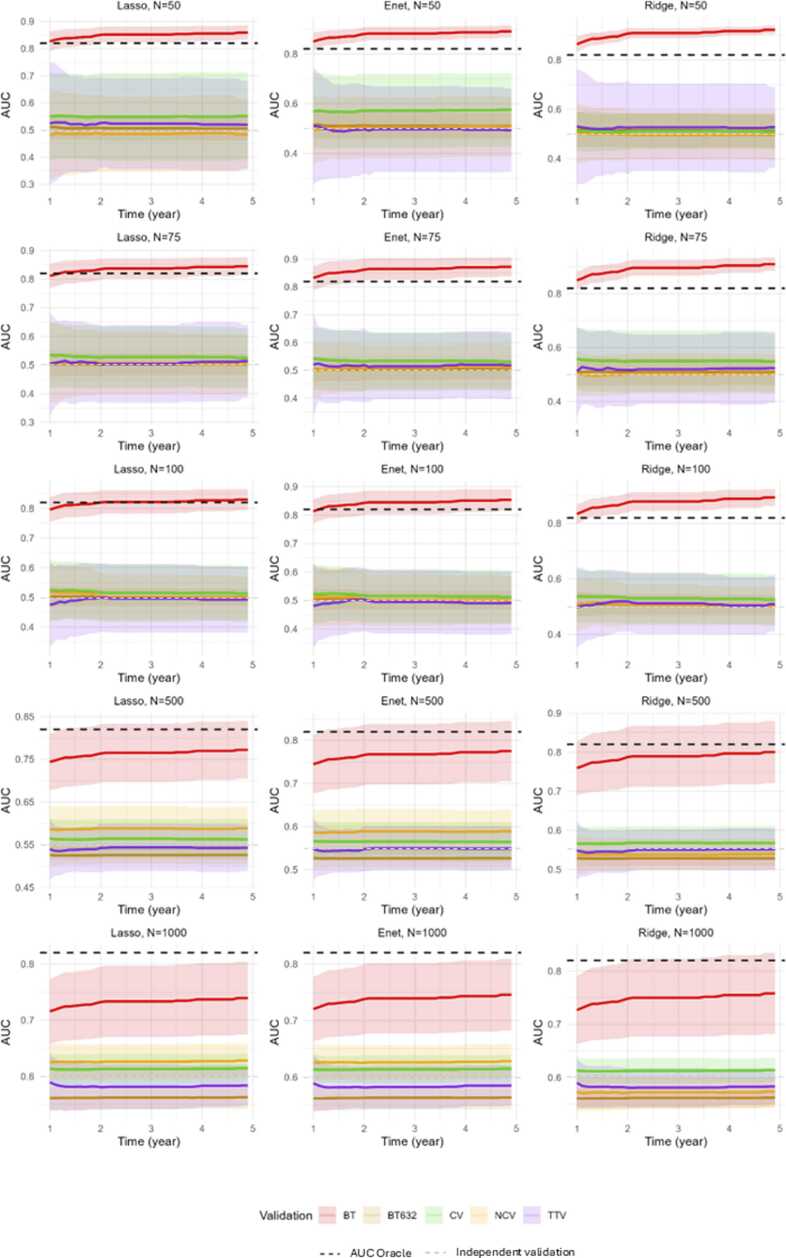
AUC time-dependent discrimination performances by internal validation strategy according to Cox penalized selection model and sample size (n = 50, n = 75, n = 100, n = 500, n = 1000).

We represented the discriminative AUC-time dependent for Cox penalized regression according to internal validity method on Fig. 2 with their variation across the time. The AUC(t) between 1 and 5 years were stable for all the internal validation strategies according to the scenarios of simulation with the variation of sample size (n = 50 to n = 1000) and the model development hyperparameter (α=0.05–0.95).

The conventional bootstrap method demonstrated higher AUC(t) performance in simulations. The discriminative performance for very low sample setting (n = 50 to n = 100) surpassed that of the oracle model, indicating a tendency towards hyper-optimistic results. In very low sample size setting (n = 50 to n = 100), the 3-years AUC of the train-test validation, 0.632 + bootstrap and nested cross-validation methods were close to 0.5. The discriminative performance of independent set validation were close to 0.5. As the sample size increased from n = 500 to n = 1000, the tendency for optimism bias in the conventional bootstrap method was less important and statistically over the other methods according to the 3-years AUC (SD) represented on Table 1. The discriminative independent set validation approach for sample size from n = 500 to n = 1000.

**Table 1:** Three-year AUC according to internal validation strategy and penalized model.

						**Sample size**					
**Validation**	**Regularization**	**50**		**75**		**100**		**500**		**1000**	
		**AUC (SD)**	**C-Index (SD)**	**AUC (SD)**	**C-Index (SD)**	**AUC (SD)**	**C-Index (SD)**	**AUC (SD)**	**C-Index (SD)**	**AUC (SD)**	**C-Index (SD)**
TTV	Lasso	0.52 (0.17)	0.52 (0.13)	0.50 (0.14)	0.51 (0.10)	0.50 (0.12)	0.50 (0.09)	0.54 (0.06)	0.54 (0.05)	0.58 (0.04)	0.57 (0.03)
	Enet	0.50 (0.17)	0.50 (0.15)	0.51 (0.12)	0.52 (0.10)	0.50 (0.11)	0.50 (0.08)	0.55 (0.05)	0.54 (0.05)	0.58 (0.04)	0.57 (0.03)
	Ridge	0.53 (0.18)	0.52 (0.14)	0.52 (0.13)	0.52 (0.10)	0.51 (0.11)	0.51 (0.08)	0.55 (0.05)	0.54 (0.05)	0.58 (0.04)	0.57 (0.03)
BT	Lasso	0.85 (0.03)	0.81 (0.03)	0.84 (0.04)	0.79 (0.04)	0.82 (0.04)	0.77 (0.04)	0.77 (0.07)	0.72 (0.06)	0.73 (0.06)	0.69 (0.05)
	Enet	0.88 (0.03)	0.83 (0.03)	0.87 (0.04)	0.81 (0.04)	0.84 (0.04)	0.79 (0.04)	0.77 (0.07)	0.72 (0.06)	0.74 (0.06)	0.70 (0.05)
	Ridge	0.91 (0.02)	0.86 (0.03)	0.90 (0.03)	0.84 (0.03)	0.88 (0.03)	0.82 (0.04)	0.79 (0.08)	0.74 (0.07)	0.75 (0.07)	0.71 (0.06)
BT632 +	Lasso	0.51 (0.05)	0.51 (0.04)	0.50 (0.04)	0.50 (0.03)	0.50 (0.03)	0.50 (0.02)	0.53 (0.02)	0.52 (0.02)	0.56 (0.02)	0.55 (0.02)
	Enet	0.51 (0.05)	0.51 (0.04)	0.50 (0.04)	0.50 (0.03)	0.50 (0.03)	0.50 (0.03)	0.53 (0.02)	0.52 (0.02)	0.56 (0.02)	0.55 (0.02)
	Ridge	0.51 (0.07)	0.51 (0.06)	0.51 (0.05)	0.51 (0.04)	0.51 (0.04)	0.50 (0.03)	0.53 (0.02)	0.52 (0.02)	0.56 (0.02)	0.55 (0.02)
CV	Lasso	0.55 (0.16)	0.52 (0.07)	0.53 (0.11)	0.52 (0.08)	0.52 (0.10)	0.52 (0.08)	0.56 (0.04)	0.57 (0.05)	0.61 (0.03)	0.60 (0.02)
	Enet	0.57 (0.15)	0.54 (0.07)	0.53 (0.11)	0.53 (0.08)	0.52 (0.09)	0.51 (0.08)	0.57 (0.05)	0.57 (0.06)	0.61 (0.03)	0.60 (0.02)
	Ridge	0.51 (0.07)	0.50 (0.11)	0.55 (0.11)	0.54 (0.08)	0.53 (0.09)	0.53 (0.08)	0.57 (0.06)	0.57 (0.06)	0.61 (0.02)	0.60 (0.02)
NCV	Lasso	0.48 (0.14)	0.49 (0.08)	0.51 (0.11)	0.50 (0.06)	0.50 (0.08)	0.51 (0.05)	0.59 (0.05)	0.58 (0.04)	0.63 (0.03)	0.62 (0.03)
	Enet	0.50 (0.12)	0.50 (0.08)	0.51 (0.08)	0.51 (0.07)	0.50 (0.09)	0.50 (0.08)	0.59 (0.05)	0.58 (0.04)	0.63 (0.03)	0.62 (0.03)
	Ridge	0.49 (0.10)	0.50 (0.09)	0.50 (0.07)	0.50 (0.06)	0.51 (0.07)	0.51 (0.07)	0.54 (0.04)	0.54 (0.05)	0.57 (0.03)	0.57 (0.04)

Abbreviation: BT632 + = 0.632 + Bootstrap; BT = Conventional Bootstrap;; CV = K-fold cross-Validation; NCV = Nested cross-validation; TTV = Train-test validation

* We represented 3 years AUC with (SD)

### 3.2 Calibration performance

We represented on Table 2 the 3 years-IBS according to scenario, regularization and internal validation method. The oracle 3 years integrated Brier Score was 0.159.

**Table 2:** Three-year IBS according to internal validation strategy and penalized model.

		**Sample size**				
		50	75	100	500	100
**Validation**	**Regularization**	IBS (SD)*				
TTV	Lasso	0.156 (0.033)	0.162 (0.027)	0.165 (0.022)	0.166 (0.010)	0.167 (0.008)
	Enet	0.156 (0.034)	0.161 (0.028)	0.165 (0.022)	0.166 (0.009)	0.167 (0.008)
	Ridge	0.156 (0.034)	0.162 (0.027)	0.165 (0.022)	0.166 (0.009)	0.167 (0.008)
BT	Lasso	0.131 (0.015)	0.136 (0.015)	0.139 (0.012)	0.144 (0.011)	0.149 (0.008)
	Enet	0.128 (0.016)	0.132 (0.013)	0.136 (0.013)	0.144 (0.012)	0.148 (0.009)
	Ridge	0.123 (0.013)	0.127 (0.014)	0.131 (0.012)	0.142 (0.012)	0.147 (0.010)
BT632 +	Lasso	0.150 (0.016)	0.153 (0.014)	0.157 (0.011)	0.160 (0.006)	0.160 (0.004)
	Enet	0.150 (0.016)	0.153 (0.014)	0.157 (0.011)	0.160 (0.010)	0.160 (0.004)
	Ridge	0.150 (0.016)	0.153 (0.014)	0.157 (0.011)	0.160 (0.006)	0.160 (0.004)
CV	Lasso	0.126 (0.014)	0.143 (0.015)	0.149 (0.011)	0.151 (0.007)	0.157 (0.004)
	Enet	0.125 (0.015)	0.142 (0.014)	0.148 (0.012)	0.149 (0.007)	0.157 (0.004)
	Ridge	0.141 (0.015)	0.142 (0.014)	0.148 (0.011)	0.146 (0.006)	0.157 (0.004)
NCV	Lasso	0.151 (0.019)	0.154 (0.016)	0.159 (0.013)	0.158 (0.006)	0.157 (0.005)
	Enet	0.152 (0.019)	0.154 (0.016)	0.157 (0.014)	0.158 (0.006)	0.157 (0.004)
	Ridge	0.154 (0.018)	0.155 (0.016)	0.158 (0.013)	0.160 (0.007)	0.159 (0.004)

Abbreviation: BT632 + = 0.632 + Bootstrap; BT = Conventional Bootstrap;; CV = K-fold cross-Validation; NCV = Nested cross-validation; TTV = Train-test validation

* We represented 3 years IBS with (SD)

The choice of penalized selection method had only a minor impact on calibration, with slight variation across different scenarios. Internal validation influenced calibration in a manner similar to its effect on discriminative performance. The train-test method exhibited the poorest performance with higher 3years IBS (SD) than the other methods for all the sample size from n = 50 to n = 1000 and for all the regularization of penalized regression. Calibration performance in bootstrap validation declined with increasing sample size, highlighting its susceptibility to hyper-optimism, particularly in high-dimensional, low-sample-size settings (n = 50 to n = 100). The K-fold cross-validation method exhibits a more discrete variation in performance, showing a decline as the sample size increases from n = 100 to n = 500. In contrast, the nested cross-validation method maintains stable calibration performance across different sample sizes (n = 50 to n = 1000).

### 3.3 Variance and stability

The homogeneity test of the variance of the 3-years AUC comparing all the internal validation strategies according to regularization of penalized regression and sample size were reported on eTable 3 and were for all the variation of regularization and sample size statistically significant (p < 0.05). The Fisher adjusted pairwise comparison according all scenarios of simulation were represented on eTable 4 with a less important variance for k-fold cross-validation and nested cross-validation approaches (p > 0.05) in sample size (n = 50,75,100,500) for both Lasso-like and Elastic-net regularization. The train-test validation approach demonstrated a higher variance compared to other internal validation strategies, underscoring its instability. In contrast, k-fold cross-validation exhibited a stable performance over time and across different regularization methods. Nested cross-validation, however, exhibited variability depending on the specifics of the model development regularization but consistent across the sample size excepted for Ridge-like regression with n = 1000 (supplementary materiel: eTable 4) with higher variance for Nested cross-validation approach.

**Fig. 3:**
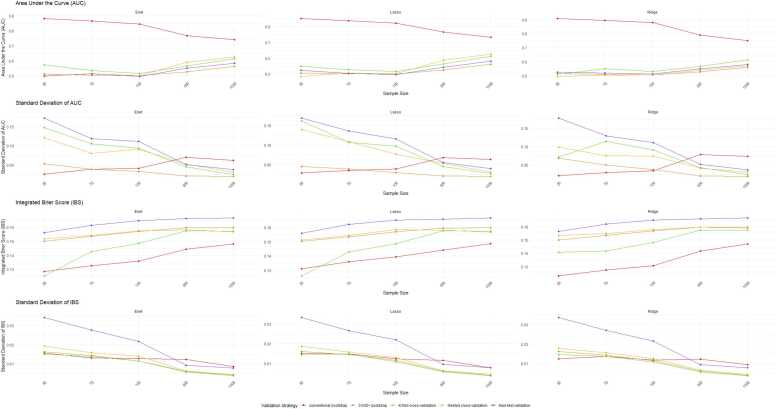
Combined plot representing The 3 years AUC, 3 years IBS and their standard deviation variation according to sample size, internal validation method and regularization.

We summarized the performance of the internal validation process in a combined plot (Fig. 3) according to scenario sample size, regularization of penalized regression, internal validation approaches, 3 year-AUC and 3 years IBS. The performance assessment and their variation are described as most consistent for the K-fold cross-validation and nested-cross validation across the regularization of penalized regression and the variation of sample size n = 50 to n = 1000.

### 3.4 Application on SCANDARE dataset

We estimated the coefficients using the n = 76 samples with the lasso-like approach (*a*= 0.95) for selection of variables. We included a matrix of 4 clinical variables (Age, Sex, HPV status, TNM staging) and 14928 transcripts normalized in log-TPM scale for model development.

**Fig. 4:**
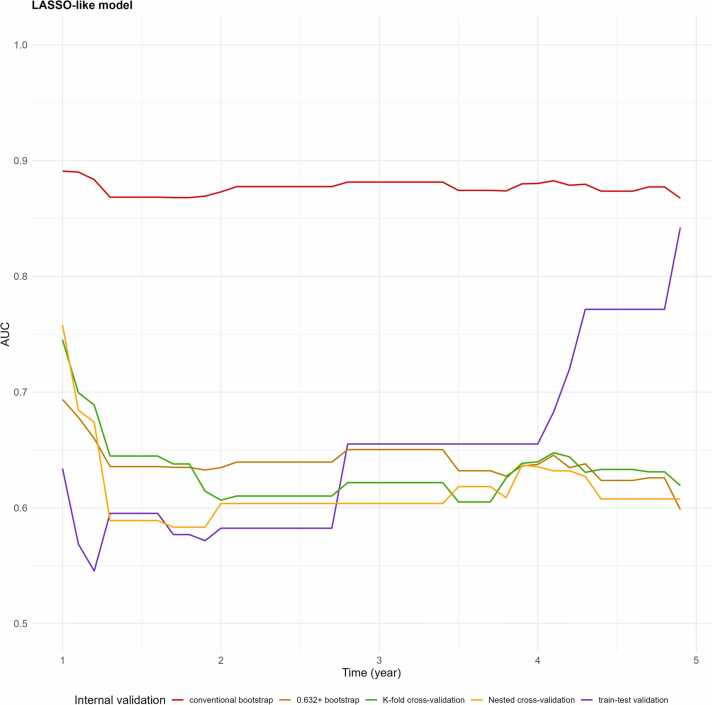
AUC(t) according to internal validation method of the lasso-like model using SCANDARE dataset.

We represented the AUC(t) of internal validation of the lasso-like model in Fig. 4. Among the validation strategies, the conventional bootstrap method demonstrated the highest discriminative performance, followed by the k-fold cross-validation, and then nested cross-validation. The Train-test validation described an instable variation of the AUC over the time.

We represented the 3-years IBS of the lasso-like model for the five previous methods of internal validation on the Table 3. The conventional bootstrap and the k-fold cross-validation showed the best calibration performance. The Train-test method is prone to a greater discrepancy between the predicted event probabilities and actual observations.

**Table 3:** Three-year IBS according to internal validation strategy and penalized model of SCANDARE lasso-like model.

**Validation**	3-year IBS *
**TTV**	0.187
**Boot**	0.144
**0.632 + Boot**	0.167
**CV**	0.154
**Nested CV**	0.167

Abbreviation: 0.632 +Boot = 0.632 + Bootstrap; Boot = Conventional Bootstrap; CV = K-fold cross-Validation; TTV = Train-test validation

The non-zero coefficients and their associated variables were obtained by applying a penalized Cox regression (LASSO) model to the SCANDARE head and neck cancer cohort. Selection was performed at the lambda value corresponding to minimum cross-validated error (λ₁se), targeting the prediction of disease-free survival at 3 years. Only variables with non-zero coefficients at this threshold are considered part of the prognostic signature. These selected variables and their corresponding non-zero coefficients are detailed in supplementary material (eTable 5), representing the LASSO-derived multi-marker signature applied for prediction of disease-free survival at 3 years in the SCANDARE cohort including 29 transcriptomics covariables.

## 4 Discussion

In this study, we aimed to compare different internal validation strategies in scenarios where the number of predictors vastly exceeds the sample size, focusing on right-censored time-to-event outcomes using all the comprehensive information for analysis. These complexities make it challenging to accurately assess both discriminative and calibration performance. We demonstrated the impact of the validation method on predictive performances and the potential risk of overestimating model performance prior to independent validation set. This study highlights the necessity of standardizing statistical approaches and collaboration in translational research to ensure the most accurate and reliable prognostic models for use in clinical practice [27], [28].

### 4.1 Parameters of simulation

In this simulation study, we performed penalization regression for prediction model development especially in high dimensional settings as recommended in statistical literature [29], [30], [31]. Some model development methods include evaluating model performance through internal validation, which is sometimes inseparable from the model's optimization and development phase. When selection occurs prior to validation, there is a risk of selection bias that must be taken into consideration [32]. The final model must be integrated using appropriate methods and metrics to prevent from optimism bias. We chose to independently simulate clinical and transcriptomic variables, a strategy that may reduce the overall performance of final model and introduce some instability in regularization methods such as ridge regression. This approach was deliberately adopted to highlight the challenges of variable selection while preserving the inherent complexity of high-dimensional data. In this simulation study, we balanced the most current parameters for internal validation methods using a train-test split ratio of 70/30 % or K = 5 folds in the k-fold cross-validation while considering the computational cost of the simulation process. These choices reflect widely accepted practices in biostatistics and machine learning, where a 70 % training and 30 % testing split is commonly used to optimize model learning and evaluation [33], and 5-fold cross-validation [34] provides a robust yet computationally feasible method for performance assessment.

### 4.2 Performance metrics and bias

We established criteria for metric evaluation using a time-dependent approach to assess the stability of internal method evaluation. The first criterion involves a discriminative approach using the cumulative/dynamic AUC as recommended by Uno et al. [22], Viallon et al. [23] and Kamarudin et al. [35]. We chose to use the AUC(t) and the C-index [36] metrics as discriminative approaches, the AUC(t) provides a dynamic assessment of the model’s discriminative ability at clinically relevant time points. The second criterion was a calibration method using the integrated Brier Score [21], [22] to measure prediction errors over time. We aimed to measure the optimism bias using the oracle performances, which represents the best possible model performances with all true coefficients used for simulation process. This oracle model serves as a benchmark that should not be surpassed. Additionally, we employed an independent validation method to assess the reliability of internal validation strategies, ensuring they do not overestimate model performance. We also evaluated how closely the performance of internal validation methods aligns with that of the independent validation process to determine how far the internal validation process could be compared with a different dataset. To ensure a direct comparison between the internal validation strategies the hyperparameters *a* of glmnet package and $\lambda_{\left(1\cdot \mathrm{SE}\right)}$ of the model was priorly fixed except for the nested cross-validation approach, which was specifically used to assess the extent of optimism bias introduced by hyperparameter estimation and ensure an unbiased evaluation of model performance.

### 4.3 Internal validation methods

One of the challenges in evaluating the performance of a statistical model is the potential dependence on random partitioning. Resampling methods help to mitigate this dependence and the instability associated with random partitioning. However, the optimism bias is not addressed in the same way across different resampling methods. Authors confirmed recently that resampling approaches perform better to internally validate the association between a time-dependent binary indicator and a time-to-event outcome [37].

With this study we confirmed the inadequate performance in high-dimensional settings of train-test validation method. When the data is divided in these contexts, there is an increased risk that the model’s λ parameters are trained on insufficient data, resulting in a strong dependency on the data splitting point. We chose to divide into train data and test data with a 70/30 % ratio which is the most current in the literature. The randomization of the sample in training set and test set itself can lead to an important variability of the performances. This simulation study confirmed the negative impact of this method using time-to event endpoint, as previously described in the literature [7], yet still commonly employed in current research which suffers from larger instability in high-dimensional setting.

The conventional bootstrap method consistently demonstrates inherent optimism, irrespective of the regularization method employed [9], [11]. This optimism bias is especially pronounced in very low setting of the sample size. When α is low, the elastic-net regularization leans more towards ridge regression, which tends to include more non-zero coefficients in the model, potentially increasing this optimism bias with a non-reproducible assessment of the performance on independent validation approach. Adding clinical variables on the Cox penalized model reduce the optimism bias but not sufficiently to avoid an important gap between the performance evaluation of internal and independent validation particularly in very low sample size setting. When using the 0.632 + bootstrap estimator, the performance of internal validation seemed to be inferior of the independent validation process. We confirmed the risk of underfitting described by Iba et al. [12] with a setting of > 10 variables per event in the high dimensional space in this particular setting of experiment. The underfitting remains problematic considering the difficulty to develop a model with good predictive performance.

The k-fold cross-validation is a robust method for assessing model performance that helps reduce the risk of overfitting. It provides stable and reliable performance estimates and is particularly recommended for logistic regression and other binary outcome models [38]. Recently, k-fold cross-validation approaches were described as reference for binary variable using time-to event endpoint [37]. Unlike other validation techniques that might be prone to optimistic bias, k-fold cross-validation effectively splits the data multiple times, providing an average assessment of model performance across different subsets. This iterative approach helps in capturing the true variability of the dataset, leading to consistent and reducing the optimism bias [14]. Extensive research has demonstrated that k-fold cross-validation [13], [15], [39], particularly in the context of high-dimensional data, offers a balanced evaluation by preventing the model from becoming overly tailored to any single training subset. This stability is crucial for developing predictive models that generalize well to new, unseen data.

Different implementation of K-fold cross-validation are described in the literature as the stratified cross-validation when unbalanced variable could affect the modelling [40] or the repeated cross-validation (RCV) [41] to improve the stability of the predictive performance. The RCV amplifies these benefits by iterating the cross-validation process multiple times, which helps in stabilizing the performance estimates. The leave-one-out (LOOV) [38], [42] validation is a special case of k-fold cross-validation where k = n, but it suffers from high computational cost in high-dimensional settings and therefore was not tested in this study. Among the various methods evaluated, the nested cross-validation approach [36], which performs both model development and internal validation simultaneously, is considered as a feasible strategy that demonstrates satisfactory stability in high-dimensional settings with TTE with no significant difference between the variance with K-fold cross-validation especially in Lasso-like and Elastic-net regularization in very low settings (n = 50, n = 75, n = 100). To avoid overfitting in model development, it’s preferable to treat the selection process as an integral part of the model fitting process and performance evaluation on every subsample [43]. The k-fold cross-validation approaches offer stability using different regularization pathway of penalized regression. This approach reduces the variance associated with a single cross-validation run and mitigates the risk of overfitting. As reported on Fig. 3, The performance assessment and their variation are described as most consistent for k-fold cross-validation and nested-cross validation.

Performance in settings with very small sample sizes may be poor, highlighting the inherent challenge of achieving robust independent validation for models developed under such constraints.

Key points:

1) K-fold cross validation produced the most stable internal validation results
2) Nested cross-validation could be performed as an alternative
3) Conventional bootstrap and train-test validation have a risk of overfitting or instability
4) 0.632 +Bootstrap estimator have a risk of underfitting
5) Independent validation of model developed with very low sample setting is hazardous after development using high dimensional setting

Improving the predictive performance of multi-omics models fundamentally depends on the methods used to integrate diverse data types, yet it should not be contingent on the choice of predictive performance evaluation strategy. In high-dimensional settings, where the number of predictors vastly exceeds the number of observations, penalized regression methods like lasso, ridge, and elastic-net are commonly used to manage overfitting and enhance variable selection. However, these methods can sometimes exclude clinically relevant variables if they are penalized alongside other predictors. Integrating the omics information using adaptative penalized regression incorporating appropriate weights especially for clinical predictor can increase power to identify the prognostic covariates and improve risk prediction [44] or hierarchical integration as priority Lasso [45] or IPF-Lasso [46] (Integrative *L*1-Penalized regression with penalty factors). The clinical variables in high-dimensional selections appears to significantly improve model performance and reduce the risk of overfitting [9]. Clinical variables often hold substantial predictive power and relevance, and their inclusion with a different penalties ensures that these important predictors are retained in the model, regardless of the penalization applied to other high-dimensional data. Adaptative methods and hierarchical regularization aim to preserve their predictive influence leading to more robust and clinically interpretable models, improving the overall predictive accuracy and stability of the model. This approach leverages the inherent value of clinical variables while effectively managing the inclusion of additional high-dimensional data through regularization techniques.

### 4.4 Perspectives

We confirmed in this study the importance of resampling methods, such as the implementation of k-fold cross-validation, to develop and assess the predictive performance of prognosis models integrating clinical and continuous variables. These findings could be further evaluated using different distribution settings of covariables and coefficients in simulation processes, with applications across various cancer types. Future studies should aim to validate these results on larger patient cohorts to enhance the generalizability and robustness of the conclusions. Nested cross-validation offers a good tradeoff between bias and stability by developing and assessing the model within the same framework. These framework have to be developed and implemented using different integration pathway as hierarchical integration, Bayesian approach and multi-source integration to confirm these stability with different setting. A key challenge in improving modeling quality with multi-omics data lies in the creation and implementation of collaborative databases as The Cancer Genome Atlas (TCGA) Research Network [47] that are continuously enriched over time. This approach can help address the high-dimensionality issue by increasing the effective sample size, thereby enhancing model robustness and generalizability of personalized prognosis models developed for clinical management.

### 4.5 Limits

We aimed to fix the parameters of the simulation using the original dataset of 76 patients to perform a realistic simulation. Nevertheless, this relatively small sample size poses limitations, including potentially reduced representativeness and limited statistical power to fully capture the diversity of the real-world patient population. While our objective was not to perfectly replicate real-world distributions, we acknowledge that this limitation could impact the generalizability of our findings to populations with distributions similar to those in our simulation study. Nevertheless, with this approach we did not included some parameters in our scenario as the censoring variation impact on the validation strategies or the optimization of the K parameters for the K-fold cross-validation approach. Some parameters variation of the simulation K-fold variation or the inner loop variation affected computational time and increased the stability of the analyses and were not reported. Moreover, the censoring probability of the event was not assessed on different setting. When combining different feature selection methods, the independent validation performance was inconsistent. This variability is likely due to the challenge of selecting non-zero coefficients and accurately estimating prognostic coefficients when the covariate matrix is high-dimensional and not prefiltered using statistical procedures such as univariable selection. Prior dimensionality reduction is critical to improve model stability and generalizability in high-dimensional omics data analyses [48]. However, as the sample size increased, the signal detected during both the development and internal validation processes was more reliably confirmed through independent validation.

## 5 Conclusions

In our study, we aimed to compare different methods of internal validation for prognostic models to contribute to the reproducibility of translational research. Our findings suggest that, for penalized models, nested cross-validation offers a balanced approach between model development and internal validation, effectively mitigating optimism bias and providing controlled stability. Repeated k-fold cross-validation emerges as a viable alternative maintaining limited optimism bias and stability prior to independent validation.

While these findings underscore the strengths of variations of cross-validation methods in high-dimensional, time-to-event outcome settings, it is important to acknowledge the limitations of our study. For instance, the simulation scenarios may not capture all complexities of real-world data, and the performance of the evaluated methods could vary with different datasets or settings.

We recommend the application of k-fold cross-validation or nested-cross validation as internal validation approaches for penalized models in high-dimensional contexts, while emphasizing the need for external validation to confirm generalizability with different setting.

## CRediT authorship contribution statement

**Constance LAMY:** Writing – review & editing, Visualization, Validation, Resources, Project administration. **Maud KAMAL:** Writing – review & editing, Visualization, Resources, Project administration. **Ladidi AHMANACHE:** Writing – review & editing, Visualization, Validation, Resources. **Jerzy KLIJANIENKO:** Writing – review & editing, Visualization, Validation, Resources. **VACHER sophie:** Writing – review & editing, Visualization, Validation, Resources. **Célia DUPAIN:** Writing – review & editing, Visualization, Validation, Resources, Project administration, Methodology. **LE TOURNEAU christophe:** Writing – review & editing, Writing – original draft, Visualization, Validation, Supervision, Resources, Project administration, Methodology, Conceptualization. **Antoine DUBRAY-VAUTRIN:** Writing – review & editing, Writing – original draft, Visualization, Validation, Resources, Methodology, Investigation, Formal analysis, Data curation, Conceptualization. **Olivier CHOUSSY:** Writing – review & editing, Visualization, Validation, Project administration. **Nicolas SERVANT:** Writing – review & editing, Visualization, Validation, Resources. **Mullaert Jimmy:** Writing – review & editing, Writing – original draft, Visualization, Validation, Supervision, Project administration, Methodology, Formal analysis, Conceptualization. **Victor GRAVRAND:** Writing – review & editing, Visualization, Validation, Resources, Methodology, Investigation, Formal analysis. **Marret Grégoire:** Writing – review & editing, Visualization, Validation, Resources, Investigation.

## Ethics statement

Not applicable. Consent for publication Not applicable.

## Declaration of Competing Interest

The authors, along with all co-authors(Antoine DUBRAY-VAUTRIN, Victor GRAVRAND, Grégoire MARRET, Constance LAMY, Jerzy KLIJANIENKO, Sophie Vacher, Ladidi AHMANACHE, Maud KAMAL, Olivier CHOUSSY, Nicolas SERVANT, Célia DUPAIN, Christophe LE TOURNEAU, Jimmy MULLAERT), declare no conflicts of interest.

## Data Availability

The parameters, functions, codes for data generation and results of the current study are available in the GitHub repository using the link: https://github.com/adubrayv/Internal_validation.git

## Footnotes

Appendix A Supplementary data associated with this article can be found in the online version at doi:10.1016/j.csbj.2025.08.035.
