# Supplementary material for: Internal validation strategy for high dimensional prognosis model: A simulation study and application to transcriptomic in head and neck tumors
Source: Comput Struct Biotechnol J. 2025 Sep 3;27:3792–802. doi: 10.1016/j.csbj.2025.08.035 (PMC12451366; doi:10.1016/j.csbj.2025.08.035)
Supplement: Supplementary file 1 — Supplementary material [file mmc1.docx]

**eTable 1 : Clinical covariates β Coefficients**

| **Variable** | **Beta (95% CI)** |
| --- | --- |
| Age (/year) | 0.02 (0.00, 0.05) |
| Sex (Female) | -0.82 (-1.58, -0.07) |
| TNM | 0.32 (0.03, 0.61) |
| HPV associated | -0.46 (-1.31, 0.40) |
