# Supplementary material for: Internal validation strategy for high dimensional prognosis model: A simulation study and application to transcriptomic in head and neck tumors
Source: Comput Struct Biotechnol J. 2025 Sep 3;27:3792–802. doi: 10.1016/j.csbj.2025.08.035 (PMC12451366; doi:10.1016/j.csbj.2025.08.035)
Supplement: Supplementary file 2 — Supplementary material [file mmc2.docx]

**eTable 2: Percentage of non-convergence models according to validation method**

|  | **Percentage of non convergence *** | | | | |
| --- | --- | --- | --- | --- | --- |
| **Model** | **N = 100** | **N = 500** | | **N = 1000** | |
| **SV** |  | |  | |  |
| Lasso-like | 3 | 3 | | 1 | |
| Enet | 3 | 4 | | 3 | |
| Ridge-like | 9 | 1 | | 1 | |
| **Boot** |  |  | |  | |
| Lasso-like | 18 | 9 | | 4 | |
| Enet | 14 | 11 | | 4 | |
| Ridge-like | 11 | 5 | | 2 | |
| **0.632+Boot** |  |  | |  | |
| Lasso-like | 0 | 0 | | 0 | |
| Enet | 0 | 0 | | 0 | |
| Ridge-like | 0 | 0 | | 0 | |
| **CV** |  |  | |  | |
| Lasso-like | 12 | 0 | | 0 | |
| Enet | 7 | 0 | | 0 | |
| Ridge-like | 6 | 0 | | 0 | |
| **Nested CV** |  |  | |  | |
| Lasso-like | 0 | 0 | | 0 | |
| Enet | 0 | 0 | | 0 | |
| Ridge-like | 0 | 0 | | 0 | |
| *Abbreviation: 0.632+boot = 0.632+ Bootstrap; Boot = Conventional Bootstrap; CV = Cross-Validation; TTV = Train-test validation* | | | | | |
