# Supplementary material for: Internal validation strategy for high dimensional prognosis model: A simulation study and application to transcriptomic in head and neck tumors
Source: Comput Struct Biotechnol J. 2025 Sep 3;27:3792–802. doi: 10.1016/j.csbj.2025.08.035 (PMC12451366; doi:10.1016/j.csbj.2025.08.035)
Supplement: Supplementary file 3 — Supplementary material [file mmc3.docx]

**eTable 3 : Homogeneity test comparing the variance between validation strategies according to regularization and sample size**

| **Regularization** | **Sample size** | **p-value*** |
| --- | --- | --- |
| Lasso | 50 | 1.37e-54 |
|  | 75 | 1.88e-43 |
|  | 100 | 5.05e-43 |
|  | 500 | 2.47e-25 |
|  | 1000 | 1.22e-30 |
| Enet | 50 | 4.03e-45 |
|  | 75 | 9.66e-35 |
|  | 100 | 5.19e-37 |
|  | 500 | 7.67e-24 |
|  | 1000 | 5.76e-31 |
| Ridge | 50 | 6.62e-52 |
|  | 75 | 1.73e-43 |
|  | 100 | 7.79e-38 |
|  | 500 | 2.75e-34 |
|  | 1000 | 4.25e-48 |

*Homogeneity test of variance using Bartlett test
