# Supplementary material for: Internal validation strategy for high dimensional prognosis model: A simulation study and application to transcriptomic in head and neck tumors
Source: Comput Struct Biotechnol J. 2025 Sep 3;27:3792–802. doi: 10.1016/j.csbj.2025.08.035 (PMC12451366; doi:10.1016/j.csbj.2025.08.035)
Supplement: Supplementary file 4 — Supplementary material [file mmc4.docx]

**eTable 4 : Fisher test for adjusted pairwise comparison of the variance between the internal validation strategies**

| **Regularization** | **Sample size** | **Pairwise comparison** | **Adjusted p-value (Bonferroni)** |
| --- | --- | --- | --- |
| Lasso | 50 | TTV vs BT | 1.80e-12 |
|  |  | TTV vs BT632 | **2.09e-01** |
|  |  | TTV vs CV | **1.00e+00** |
|  |  | TTV vs NCV | **1.00e+00** |
|  |  | BT vs BT632 | 1.04e-18 |
|  |  | BT vs CV | 2.50e-10 |
|  |  | BT vs NCV | 1.70e-15 |
|  |  | BT632 vs CV | **1.12e-01** |
|  |  | BT632 vs NCV | **1.00e+00** |
|  |  | CV vs NCV | **1.00e+00** |
| Lasso | 75 | TTV vs BT | 7.04e-16 |
|  |  | **TTV vs BT632** | **1.73e-01** |
|  |  | **TTV vs CV** | **1.00e+00** |
|  |  | **TTV vs NCV** | **1.00e+00** |
|  |  | BT vs BT632 | 8.56e-24 |
|  |  | BT vs CV | 7.01e-14 |
|  |  | BT vs NCV | 1.13e-16 |
|  |  | BT632 vs CV | 2.85e-02 |
|  |  | **BT632 vs NCV** | **3.82e-01** |
|  |  | **CV vs NCV** | **1.00e+00** |
| Lasso | 100 | TTV vs BT | 1.54e-17 |
|  |  | **TTV vs BT632** | **1.90e-01** |
|  |  | **TTV vs CV** | **1.00e+00** |
|  |  | **TTV vs NCV** | **1.00e+00** |
|  |  | BT vs BT632 | 4.84e-26 |
|  |  | BT vs CV | 2.11e-18 |
|  |  | BT vs NCV | 3.86e-19 |
|  |  | **BT632 vs CV** | **5.10e-01** |
|  |  | **BT632 vs NCV** | **9.65e-01** |
|  |  | **CV vs NCV** | **1.00e+00** |
| Lasso | 500 | TTV vs BT | 2.19e-19 |
|  |  | TTV vs BT632 | 9.11e-05 |
|  |  | TTV vs CV | 2.08e-04 |
|  |  | TTV vs NCV | 1.23e-05 |
|  |  | BT vs BT632 | 8.60e-38 |
|  |  | BT vs CV | 5.25e-37 |
|  |  | BT vs NCV | 2.09e-06 |
|  |  | **BT632 vs CV** | **1.00e+00** |
|  |  | BT632 vs NCV | 7.02e-19 |
|  |  | CV vs NCV | 3.29e-18 |
| Lasso | 1000 | TTV vs BT | 2.82e-34 |
|  |  | TTV vs BT632 | 3.71e-05 |
|  |  | TTV vs CV | 6.05e-07 |
|  |  | TTV vs NCV | 3.33e-11 |
|  |  | BT vs BT632 | 3.46e-55 |
|  |  | BT vs CV | 7.08e-14 |
|  |  | BT vs NCV | 1.70e-09 |
|  |  | BT632 vs CV | 5.75e-21 |
|  |  | BT632 vs NCV | 3.16e-27 |
|  |  | **CV vs NCV** | **1.00e+00** |
| Enet | 50 | TTV vs BT | 1.57e-12 |
|  |  | **TTV vs BT632** | **1.00e+00** |
|  |  | **TTV vs CV** | **1.94e-01** |
|  |  | **TTV vs NCV** | **1.00e+00** |
|  |  | BT vs BT632 | 2.56e-15 |
|  |  | BT vs CV | 4.47e-07 |
|  |  | BT vs NCV | 3.85e-13 |
|  |  | BT632 vs CV | 8.97e-03 |
|  |  | **BT632 vs NCV** | **1.00e+00** |
|  |  | **CV vs NCV** | **1.50e-01** |
| Enet | 75 | TTV vs BT | 8.51e-15 |
|  |  | TTV vs BT632 | 1.22e-02 |
|  |  | **TTV vs CV** | **1.00e+00** |
|  |  | **TTV vs NCV** | **1.00e+00** |
|  |  | BT vs BT632 | 9.78e-27 |
|  |  | BT vs CV | 1.99e-15 |
|  |  | BT vs NCV | 2.99e-18 |
|  |  | BT632 vs CV | 1.53e-02 |
|  |  | **BT632 vs NCV** | **1.62e-01** |
|  |  | **CV vs NCV** | **1.00e+00** |
| Enet | 100 | TTV vs BT | 2.84e-21 |
|  |  | **TTV vs BT632** | **1.00e+00** |
|  |  | **TTV vs CV** | **1.00e+00** |
|  |  | **TTV vs NCV** | **1.00e+00** |
|  |  | BT vs BT632 | 1.60e-26 |
|  |  | BT vs CV | 3.76e-18 |
|  |  | BT vs NCV | 1.68e-20 |
|  |  | **BT632 vs CV** | **2.72e-01** |
|  |  | **BT632 vs NCV** | **1.00e+00** |
|  |  | **CV vs NCV** | **1.00e+00** |
| Enet | 500 | TTV vs BT | 5.94e-18 |
|  |  | TTV vs BT632 | 1.25e-05 |
|  |  | TTV vs CV | 4.76e-06 |
|  |  | TTV vs NCV | 3.63e-05 |
|  |  | BT vs BT632 | 1.45e-37 |
|  |  | BT vs CV | 3.36e-38 |
|  |  | BT vs NCV | 5.50e-06 |
|  |  | **BT632 vs CV** | **1.00e+00** |
|  |  | BT632 vs NCV | 1.71e-19 |
|  |  | CV vs NCV | 3.39e-20 |
| Enet | 1000 | TTV vs BT | 2.36e-34 |
|  |  | TTV vs BT632 | 2.25e-04 |
|  |  | TTV vs CV | 4.84e-07 |
|  |  | TTV vs NCV | 6.64e-11 |
|  |  | BT vs BT632 | 1.73e-53 |
|  |  | BT vs CV | 7.08e-14 |
|  |  | BT vs NCV | 7.11e-10 |
|  |  | BT632 vs CV | 1.25e-19 |
|  |  | BT632 vs NCV | 3.55e-25 |
|  |  | **CV vs NCV** | **1.00e+00** |
| Ridge | 50 | TTV vs BT | 7.83e-11 |
|  |  | **TTV vs BT632** | **1.00e+00** |
|  |  | TTV vs CV | 3.55e-02 |
|  |  | **TTV vs NCV** | **1.00e+00** |
|  |  | BT vs BT632 | 1.62e-11 |
|  |  | BT vs CV | 2.95e-18 |
|  |  | BT vs NCV | 9.57e-15 |
|  |  | BT632 vs CV | 8.43e-02 |
|  |  | **BT632 vs NCV** | **1.00e+00** |
|  |  | **CV vs NCV** | **1.00e+00** |
| Ridge | 75 | TTV vs BT | 1.50e-18 |
|  |  | **TTV vs BT632** | **5.62e-01** |
|  |  | **TTV vs CV** | **1.00e+00** |
|  |  | **TTV vs NCV** | **7.88e-01** |
|  |  | BT vs BT632 | 1.49e-25 |
|  |  | BT vs CV | 1.73e-13 |
|  |  | BT vs NCV | 6.41e-25 |
|  |  | BT632 vs CV | 6.94e-03 |
|  |  | **BT632 vs NCV** | **1.00e+00** |
|  |  | CV vs NCV | 1.20e-02 |
| Ridge | 100 | TTV vs BT | 6.54e-20 |
|  |  | **TTV vs BT632** | **7.20e-02** |
|  |  | **TTV vs CV** | **1.00e+00** |
|  |  | **TTV vs NCV** | **1.00e+00** |
|  |  | BT vs BT632 | 4.53e-30 |
|  |  | BT vs CV | 5.50e-19 |
|  |  | BT vs NCV | 6.78e-21 |
|  |  | BT632 vs CV | 4.32e-02 |
|  |  | **BT632 vs NCV** | **1.62e-01** |
|  |  | **CV vs NCV** | **1.00e+00** |
| Ridge | 500 | TTV vs BT | 8.30e-21 |
|  |  | TTV vs BT632 | 2.81e-09 |
|  |  | TTV vs CV | 1.41e-04 |
|  |  | TTV vs NCV | 1.35e-01 |
|  |  | BT vs BT632 | 7.43e-48 |
|  |  | BT vs CV | 5.64e-08 |
|  |  | BT vs NCV | 2.35e-29 |
|  |  | BT632 vs CV | 1.04e-23 |
|  |  | BT632 vs NCV | 3.25e-03 |
|  |  | CV vs NCV | 4.05e-10 |
| Ridge | 1000 | TTV vs BT | 4.75e-26 |
|  |  | TTV vs BT632 | 4.31e-05 |
|  |  | TTV vs CV | 1.82e-10 |
|  |  | **TTV vs NCV** | **1.00e+00** |
|  |  | BT vs BT632 | 1.35e-46 |
|  |  | BT vs CV | 1.50e-05 |
|  |  | BT vs NCV | 8.72e-31 |
|  |  | BT632 vs CV | 1.85e-26 |
|  |  | BT632 vs NCV | 6.45e-03 |
|  |  | CV vs NCV | 1.16e-13 |
