# Supplementary material for: Internal validation strategy for high dimensional prognosis model: A simulation study and application to transcriptomic in head and neck tumors
Source: Comput Struct Biotechnol J. 2025 Sep 3;27:3792–802. doi: 10.1016/j.csbj.2025.08.035 (PMC12451366; doi:10.1016/j.csbj.2025.08.035)
Supplement: Supplementary file 5 — Supplementary material [file mmc5.docx]

eTable 5 : Selected variables with non-zero coefficients estimated by lasso-like approach: SCANDARE application

| **Selected variables** | **Index** | **Coefficients** |
| --- | --- | --- |
| ENSG00000142634 | 158 | 0.0005014521 |
| ENSG00000164011 | 484 | -0.0103614049 |
| ENSG00000203817 | 921 | 0.0548400195 |
| ENSG00000117595 | 1,423 | 0.0018565470 |
| ENSG00000237380 | 2,295 | 0.0142765047 |
| ENSG00000267919 | 2,490 | 0.0177172749 |
| ENSG00000012171 | 2,862 | -0.0298938254 |
| ENSG00000113580 | 4,495 | 0.0036929272 |
| ENSG00000253522 | 4,576 | -0.0037044137 |
| ENSG00000197238 | 4,825 | 0.0009059366 |
| ENSG00000172115 | 5,580 | 0.0055181257 |
| ENSG00000168303 | 5,648 | 0.0195352552 |
| ENSG00000104725 | 6,272 | 0.0001170782 |
| ENSG00000230453 | 6,810 | 0.0570647782 |
| ENSG00000107159 | 6,841 | 0.0002639503 |
| ENSG00000266315 | 7,044 | -0.0019182908 |
| ENSG00000197958 | 7,123 | 0.0002453960 |
| ENSG00000269772 | 7,573 | 0.0211648170 |
| ENSG00000177600 | 7,885 | 0.0002219758 |
| ENSG00000011347 | 8,163 | 0.0222925190 |
| ENSG00000110347 | 8,505 | -0.0006881530 |
| ENSG00000196935 | 9,135 | 0.0062771107 |
| ENSG00000177731 | 11,618 | 0.0053812253 |
| ENSG00000264635 | 12,345 | -0.0055399614 |
| ENSG00000141404 | 12,383 | -0.0032563014 |
| ENSG00000167644 | 13,083 | 0.0006028792 |
| ENSG00000086544 | 13,147 | 0.0007123616 |
| ENSG00000105732 | 13,174 | 0.0309319774 |
| ENSG00000210107 | 14,902 | 0.0007131843 |
