# Supplementary figures and images for: Internal validation strategy for high dimensional prognosis model: A simulation study and application to transcriptomic in head and neck tumors
Source: Comput Struct Biotechnol J. 2025 Sep 3;27:3792–802. doi: 10.1016/j.csbj.2025.08.035 (PMC12451366; doi:10.1016/j.csbj.2025.08.035)

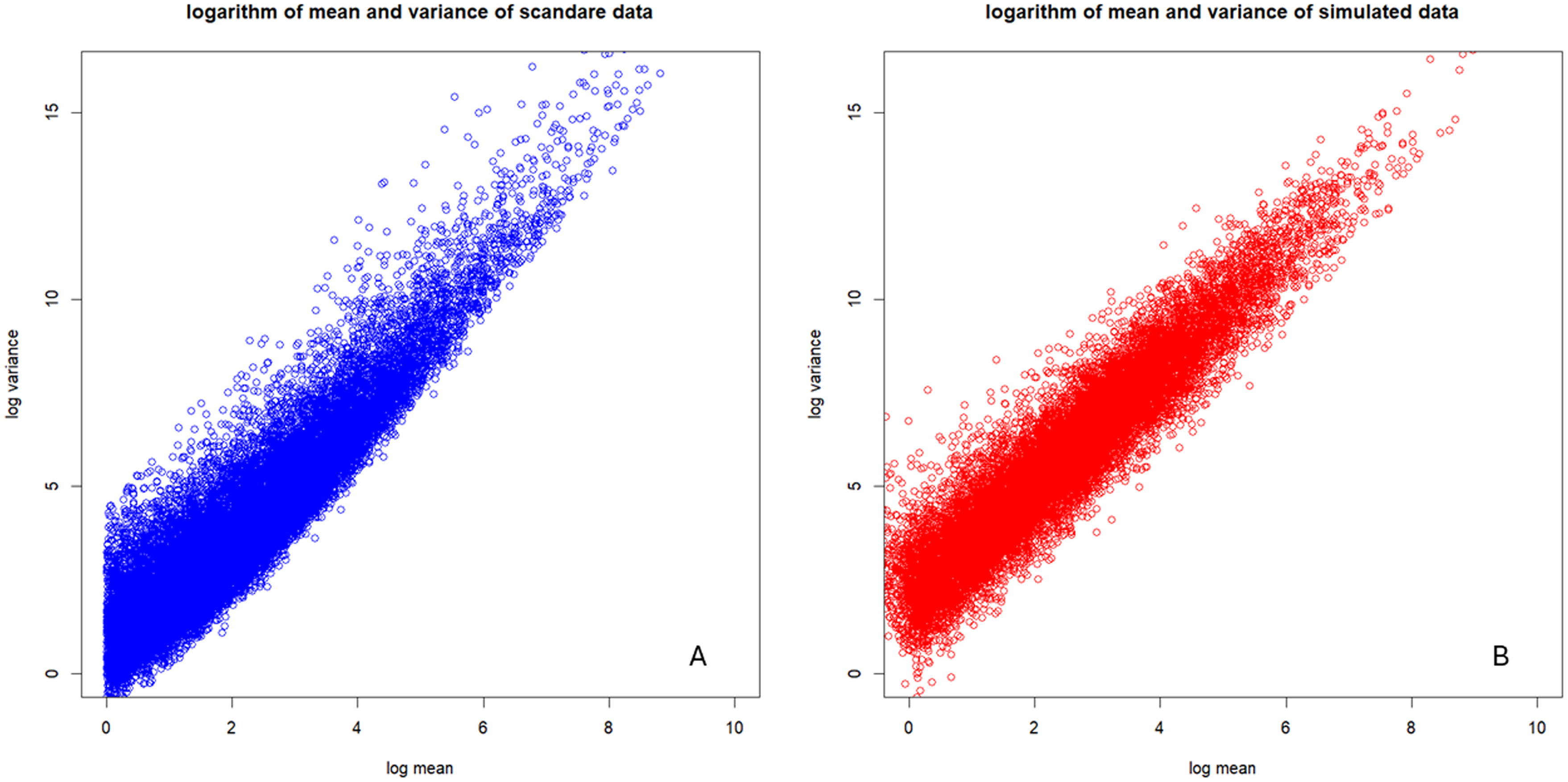

Supplement: Supplementary file 6 — Supplementary material [file mmc6.jpg]
